# Phenolic profile and biological properties of the leaves of *Ficus vasta* Forssk. (Moraceae) growing in Egypt

**DOI:** 10.1186/s12906-018-2210-0

**Published:** 2018-05-16

**Authors:** Maria Fernanda Taviano, Khaled Rashed, Angela Filocamo, Francesco Cacciola, Paola Dugo, Luigi Mondello, Carlo Bisignano, Rosaria Acquaviva, Manuela D’Arrigo, Natalizia Miceli

**Affiliations:** 1 Dipartimento di Scienze Chimiche, Biologiche, Farmaceutiche ed Ambientali, University of Messina, Polo Annunziata, Viale Annunziata, 98168 Messina, Italy; 2 Pharmacognosy Department, National Research Centre, 33 El-Bohouth st. Dokki, P.O.12622, Giza, Egypt; 3 Foundation “Prof. Antonio Imbesi”, University of Messina, Piazza Pugliatti 1, 98122 Messina, Italy; 4 Dipartimento di Scienze Biomediche, Odontoiatriche e delle Immagini Morfologiche e Funzionali, University of Messina, Via Consolare Valeria, 98125 Messina, Italy; 5 Scienze dell’Alimentazione e della Nutrizione Umana, Università Campus Biomedico di Roma, via Àlvaro del Portillo 21, 00128 Rome, Italy; 6 Chromaleont s.r.l., c/o Dipartimento di Scienze Chimiche, Biologiche, Farmaceutiche ed Ambientali, University of Messina, Polo Annunziata, Viale Annunziata, 98168 Messina, Italy; 7 Dipartimento di Scienze del Farmaco, sezione Biochimica, Viale Andrea Doria 6, 95123 Catania, Italy

**Keywords:** *Ficus vasta* Forssk., Phenolic profile, Antioxidant activity, Oxidative stress in *Escherichia coli*, Antimicrobial activity, *Artemia salina* Leach

## Abstract

**Background:**

*Ficus vasta* Forssk. (Moraceae) is traditionally used for the treatment of various ailments; nonetheless, this species has been poorly studied to date. This work aimed to characterize the phenolic profile and to evaluate the antioxidant and antimicrobial properties of a hydroalcoholic extract obtained from *F. vasta* leaves collected in Egypt.

**Methods:**

The phenolic profile of the extract was characterized by HPLC-PDA/ESI-MS. The antioxidant properties were examined by different in vitro systems: DPPH test, reducing power and metal chelating activity assays. Moreover, the ability of the extract to protect *Escherichia coli* growth and survival from H_2_O_2_-induced oxidative stress was evaluated. The potential toxicity was investigated using *Artemia salina* lethality bioassay. Finally, the antimicrobial properties against a representative set of Gram-positive and Gram-negative bacterial strains and the yeast *C. albicans* were assayed by standard methods.

**Results:**

By HPLC-PDA/ESI-MS analysis 12 compounds belonging to the groups of phenolic acids and flavonoids were identified. The extract exhibited strong radical scavenging activity in DPPH test (IC_50_ = 0.0672 ± 0.0038 mg/mL), reducing power (3.65 ± 0.48 ASE/mL) and chelating activity (IC_50_ = 0.801 ± 0.007 mg/mL). A total protection against H_2_O_2_-induced damage on *E. coli* was observed. No toxicity against *A. salina* was found (LC_50_ > 1000 μg/mL). The extract exhibited bacteriostatic activity against almost all the bacteria tested (MICs: 250–62.5 μg/mL).

**Conclusions:**

The obtained results demonstrate the potential of *F. vasta* leaves as safe sources of natural antioxidant and antimicrobial compounds.

## Background

Medicinal plants are considered as potential sources for drug development and many novel products. Nonetheless, such plants should be investigated to better understand their properties, safety, and efficiency. Currently, many researchers are looking for newer, effective, and safe antioxidants, in order to use them in foods and pharmaceutical preparations to replace the synthetic ones, which have been reported to be dangerous for human health. Medicinal plants are the major source of chemical compounds exhibiting antioxidant activity. Particularly, a direct relationship between antioxidant activity and phenolic compounds has been demonstrated in many studies [1–4].

Recently, there is an increased frequency of multiple drug resistance in human pathogenic microorganism due to indiscriminate use of commercial antimicrobial drugs commonly used to treat various diseases. Therefore, there is an interest in developing alternative antimicrobial drugs for the treatment of infections obtained from various sources such as medicinal plants. Phytochemicals have become the target of a great number of researches focused on the discovery of potentially safe and effective antimicrobials. Plant based antimicrobials either unaided or in combination with antibiotics may be an effective approach to deal with the global antimicrobial resistance. Among plant bioactive compounds, polyphenols are well documented to have microbicide activities against a great number of pathogenic bacteria [5].

The genus *Ficus* L. (Moraceae) comprises about 800 species and 2000 varieties of woody trees, shrubs and vines known as fig trees [6]. Several members of this genus are being used in folk medicine all over the world for a wide range of ailments of the gastrointestinal tract, central nervous, endocrine, and reproductive systems, as well as infectious disorders like tuberculosis, respiratory and skin diseases [7–9].

*Ficus vasta* Forssk. is a very large tree growing over 25 m tall, with spreading rounded crown. Leaves are alternate, spirally arranged, quite stiff, rough to the touch, almost circular (8–25 × 4–23 cm), margin entire, tip rounded but often with a blunt point, base rounded, heart shaped; usually glabrescent above, glabrescent, puberulous, hirsute or velutinous below [10].

This species is widespread throughout the dry north and eastern Africa, Sudan, Ethiopia, Saudi Arabia, Uganda and Tanzania. In Sudan the poultice of burned *F. vasta* leaves and barks was used as anti-tumor [11]. The leaves are traditionally used for the treatment of rheumatisms, pains and intestinal worms [12].

Although many species from the genus *Ficus* were subjected to phytochemical and pharmacological investigations, to the best of our information *F. vasta* has been poorly studied to date. Qualitative preliminary phytochemical analysis of Egyptian *F. vasta* leaves, using standard chemical tests, revealed the presence of carbohydrates, tannins, flavonoids, coumarins, and triterpenes [12]. Moreover, various phytoconstituents such as β-sitosterol, stigmasterol, lupeol, ursolic acid and some flavonoids were isolated and identified from *F. vasta* aerial parts [13]. Concerning biological activities, very few studies have been carried out on the leaves of this species [10, 14].

Thus, the present work was undertaken to characterize the phenolic profile and to investigate the antioxidant and antimicrobial properties of a hydroalcoholic extract obtained from the leaves of *F. vasta* collected in Egypt, never studied before.

## Methods

### Chemicals and reagents

LC-MS grade water (H_2_O), acetonitrile (ACN), gallic acid, catechin, naringenin, chlorogenic acid, apigenin, rutin, kaempferol and quercetin were obtained from Merck KGaA (Darmstadt, Germany). LC-MS grade acetic acid was attained from Riedel-de Haën (Seelze, Germany); methanol (MeOH) from Baker Analysed Reagent; Ferrous chloride (FeCl_2_) was obtained from Carlo Erba (Milan, Italy). Müeller Hinton Broth (MHB), Sabouraud Dextrose Agar (SDA), and Luria-Bertani (LB) broth medium were supplied from Oxoid (Basingstoke, UK), RPMI 1640 from Gibco Laboratories (Grand Island, NY). Unless indicated otherwise, all chemicals were purchased from Sigma-Aldrich (Milan, Italy).

### Plant material and extraction procedure

*Ficus vasta* leaves were collected in May from Al-Zohiriya garden, Giza, Egypt. The plant was identified by Dr. Mohammed El-Gebaly, Department of Botany, National Research Centre (NRC) and by Mrs. Tereeza Labib, consultant of Plant Taxonomy at the Ministry of Agriculture and director of Orman Botanical Garden, Giza, Egypt. A voucher specimen was deposited in the herbarium of Al-Zohiriya garden, Giza, Egypt, under accession number n° FN-2604.

The air dried and powdered *F. vasta* leaves (200 g) were extracted with 80% MeOH at room temperature several times under continuous shaking until exhaustion by maceration process. The extractive solutions were pooled, filtrated, and evaporated to dryness by rotary evaporator (40°C). The yield of *F. vasta* extract, referred to 100 g of dried leaves, was 13.00%.

### Phytochemical investigations

#### Identification of flavonoid compounds by paper chromatography

*F. vasta* hydroalcoholic extract was subjected to paper chromatography (Whatman No.1) using three different solvent systems as n-butanol:acetic acid:water (BAW 4:1:5, upper layer), 15% acetic acid, and water. By comparison with standard compounds some flavonoids were identified. Then, each band was cut, and the compounds were dissolved in a mixture of MeOH/H_2_O, purified over Sephadex LH-20 and identified by UV, ^1^H-NMR and MS analyses [15, 16].

#### Identification of phenolic compounds by HPLC-PDA/ESI-MS

HPLC-PDA/ESI-MS analyses were performed on a Prominence LC system (Shimadzu, Milan, Italy) equipped with photo diode array (PDA) and mass spectrometry (MS) (LCMS-2020, Shimadzu) detection. Data acquisition was performed by Shimadzu LabSolution software ver. 5.53.

For chromatographic separations, an Ascentis Express C18 column (15 cm × 4.6 mm I.D.) packed with 2.7 μm partially porous particles, was employed (Supelco, Bellefonte, PA, USA). The injection volume was 5 μL, and the mobile phase consisted of water/acetic acid (0.1%) at pH = 3 (solvent A) and ACN/acetic acid (0.1%) (solvent B), respectively in the following linear gradient mode: 0 min, 0% B; 5 min, 5% B; 15 min, 10% B; 30 min, 20% B; 60 min, 50% B; 70 min, 100% B; 71 min, 0% B. The mobile phase flow rate was 1.0 mL/min, and it was splitted to 0.3 mL/min prior to MS detection. PDA wavelength range was 210–400 nm and the chromatograms were extracted at 280 and 350 nm.

The extract (10 mg) was dissolved in DMSO (1 mL) and filtered through a 0.45 μm membrane filters (Whatman, Clifton, USA).

Phenolics identification was carried out by the complementary information provided by chromatographic retention times, PDA and mass spectra, and further supported by comparison to existing literature data [13].

The quantitative determination of each compound was carried out by means of the external standard method using gallic acid (λ = 270), catechin (λ = 278), naringenin (λ = 283), chlorogenic acid (λ = 325), apigenin (λ = 330), rutin (λ = 355), kaempferol (λ = 365) and quercetin (λ = 370) as reference compounds in a concentration range of 1–100 ppm. With three different concentration levels. Triplicate injections were made for each level, and a linear regression was generated. The calibration curves with the external standards were obtained using concentration (mg/L) with respect to the area obtained from the integration of the PDA peaks at a wavelength of 270 nm for benzoic acid-like, 278 nm for flavan-3-ol-like, 283 nm for flavanone-like, 325 nm for cinnamic acid-like, 330 nm for flavone-glycoside-like, 354 nm for flavonol-glycoside-like and flavanone-glycoside-like, 365 nm for flavone-like and 370 nm for flavonol-like compounds. The results were obtained from the average of three determinations and are expressed as mg/g dried extract ± percent relative standard deviation (%RSD).

### Antioxidant activity

#### Free radical scavenging activity

The free radical scavenging activity of *F. vasta* extract was evaluated using the DPPH (2,2-diphenyl-1-picrylhydrazyl) test, according to the protocol previously reported [17]. An aliquot (0.5 mL) of 80% MeOH solution containing different amounts of the extract (0.0125–0.2 mg/mL) was added to 3 mL of daily prepared methanol DPPH solution (0.1 mM). The optical density change at 517 nm was measured, 20 min after the initial mixing, with a model UV-1601 spectrophotometer (Shimadzu). Butylated Hydroxytoluene (BHT) was used as reference. The scavenging activity was measured as the decrease in absorbance of the samples versus DPPH standard solution. The results were obtained from the average of three independent experiments, and are reported as mean radical scavenging activity percentage (%) ± SD. The results are also expressed as mean 50% Inhibitory Concentration (IC_50_) ± standard deviation (SD), determined graphically by interpolation of the dose-response curve; lower IC_50_ value indicates higher antioxidant activity.

#### Measurement of reducing power

The reducing power of *F. vasta* extract was evaluated by spectrophotometric detection of Fe^3+^-Fe^2+^ transformation method, as previously reported [18]. Different amounts of the extract (0.0125–0.2 mg/mL) in 1 mL solvent were mixed with 2.5 mL of phosphate buffer (0.2 M, pH 6.6) and 2.5 mL of 1% potassium ferrycyanide [K_3_Fe(CN)_6_]. The mixture was incubated at 50 °C for 20 min. The resulting solution was cooled rapidly, mixed with 2.5 mL of 10% trichloroacetic acid, and centrifuged at 3000 rpm for 10 min. The resulting supernatant (2.5 mL) was mixed with 2.5 mL of distilled water and 0.5 mL of 0.1% fresh ferric chloride (FeCl_3_), and the absorbance was measured at 700_nm_ after 10 min; the increased absorbance of the reaction mixture indicates an increase in reducing power. As blank, an equal volume (1 mL) of water was mixed with a solution prepared as described above. Ascorbic acid and BHT were used as reference standards. The results were obtained from the average of three independent experiments, and are expressed as mean absorbance values ± SD. The reducing power was also expressed as ascorbic acid equivalent (ASE/mL); when the reducing power is 1 ASE/mL, the reducing power of 1 mL extract is equivalent to 1 μmol ascorbic acid.

#### Ferrous ions (Fe^2+^) chelating activity

The Fe^2+^ chelating activity of *F. vasta* extract was estimated by measuring the formation of the Fe^2+^*-*ferrozine complex, according to the method previously reported [18]. Briefly, different concentrations of the extract (0.0125–0.2 mg/mL) in 1 mL solvent were mixed with 0.5 mL of methanol and 0.05 mL of 2 mM FeCl_2_. The reaction was initiated by the addition of 0.1 mL of 5 mM ferrozine. Then the mixture was shaken vigorously and left standing at room temperature for 10 min. The absorbance of the solution was measured spectrophotometrically at 562 nm. The control contains FeCl_2_ and ferrozine, complex formation molecules. Ethylenediaminetetraacetic acid (EDTA) was used as reference standard The results were obtained from the average of three independent experiments and are reported as mean inhibition of the Fe^2+^*-*ferrozine complex formation (%) ± SD and IC_50_ ± SD.

#### Protective effect on *Escherichia coli* under peroxide stress

The ability of *F. vasta* extract to protect bacterial growth and survival from the oxidative stress induced by hydrogen peroxide (H_2_O_2_) was evaluated according the protocol described by Smirnova et al. [19], with some modifications. *Escherichia coli* ATCC 25922 was obtained from the Department of Scienze Chimiche Biologiche Farmaceutiche ed Ambientali, University of Messina, in-house culture collection (Messina, Italy). Bacteria were grown overnight in LB medium. The overnight suspension was centrifuged (10 min at 3500 rpm), resuspended in LB fresh medium to obtain a final optical density at 600 nm (OD_600_) = 0.1, and then grown aerobically at 37 °C with shaking at 150 rpm. In mid-log phase (OD_600_ = 0.6) bacteria were centrifuged and the OD_600_ adjusted to 0.2 value with fresh medium. The bacteria suspension was then aliquoted and *F. vasta* extract (1 mg/mL) and reference standard quercetin (0.2 mM) were added. Two control groups (Ctr), with and without H_2_O_2_ treatment, were included. After 30–40 min, when OD_600_ reached a value equal to 0.4, in order to establish the ability of *F. vasta* extract to exert protection against *E. coli* growth inhibition induced from oxidative stress bacteria were treated with H_2_O_2_ (2 mM), and the growth was monitored every 20 min for 3 h.

For survival studies, the bacteria (OD_600_ = 0.4) were exposed for 30 min to a higher concentration of H_2_O_2_ (10 mM), which caused bactericidal effect. Then an aliquot of each sample was diluted in 0.9% NaCl to obtain serial dilutions (1:10). Each sample was poured onto LB-agar plates and incubated at 37 °C; after 24 h the number of viable colonies was counted to estimate the cell survival. The percentage (%) of survival was calculated according to the formula: (colony forming units (CFU) of H_2_O_2_ treated culture/CFU of untreated Ctr) × 100 [20].

The results were obtained from the average of three independent experiments and are expressed as mean absorbance ± SD and surviving (%) ± SD for protective effect on *E. coli* growth and survival, respectively. Statistical comparisons of the data were performed by Student’s t-test for unpaired data. *P*-values lower than 0.05 were considered statistically significant.

#### *Artemia salina* lethality bioassay

The potential toxicity of *F. vasta* extract was investigated using brine shrimp (*Artemia salina* Leach) lethality bioassay, according to the method previously reported [21]. The extract was tested at different concentrations (10–1000 μg/mL). Ten brine shrimp larvae, taken 48 h after initiation of hatching in artificial seawater, were transferred to each sample vial, and artificial seawater was added to obtain a final volume of 5 mL. After 24 h of incubation at 25–28 °C, the vials were observed using a magnifying glass, and surviving larvae were counted. The assay was carried out in triplicate, and median lethal concentration (LC_50_) values were determined using the probit analysis method. Extracts with LC_50_ higher than 1000 μg/mL are considered non-toxic.

### Antimicrobial activity

#### Microbial strains and culture conditions

The following strains were used as indicators for the antimicrobial testing and were obtained from the Department of Scienze Chimiche Biologiche Farmaceutiche ed Ambientali, University of Messina (Italy), in-house culture collection: *Bacillus subtilis* ATCC 6633, *Escherichia coli* ATCC 10536, *Escherichia coli* ATCC 25922, *Listeria monocytogenes* ATCC 13932, *Pseudomonas aeruginosa* ATCC 15442, *Salmonella typhimurium* ATCC 13311, *Salmonella enterica* (Wild type), *Staphylococcus aureus* ATCC 29213, and *Staphylococcus epidermidis* ATCC 12228 were grown at 37 °C in MHB; the yeast *Candida albicans* ATCC 10231 was grown at 35 °C on SDA.

#### Antimicrobial testing

The minimum inhibitory concentration (MIC) and minimum bactericidal and fungicidal concentration (MBC and MFC) values of *F. vasta* extract were determined using the in broth microdilution method according to the protocols recommended by the Clinical and Laboratory Standards Institute [22, 23].

Cultures of bacterial strains and *C. albicans* were prepared overnight in MHB and RPMI 1640, respectively; microorganism suspensions were therefore adjusted with sterile medium to give 1 × 10^6^ for bacteria and 1 × 10^4^ CFU/mL for *C. albicans*. The extract was dissolved in dimethyl sulfoxide (DMSO) (1%) and MHB to obtain a final concentration of 1 mg/mL. Two-fold serial dilutions were prepared in a 96-well plate. The tested concentrations ranged from 500 to 0.49 μg/mL. The MIC was defined as the lowest concentration (μg/mL) of extract which completely inhibit the visible growth of microorganisms in broth after 24 h of incubation for bacteria and 48 h for *C. albicans*. All experiments were performed in triplicate on three independent days. Positive and negative controls were also included.

## Results

### Phytochemical investigations

#### Identification of flavonoid compounds by paper chromatography

Chromatographic separation of *F. vasta* extract allowed the identification of some flavonoid compounds, namely luteolin, quercetin, vitexin, quercetin-3-O-*β*-galactoside and rutin. Their structures were elucidated on the basis of, UV, ^1^H-NMR, and MS analyses. The spectral information is summarized in Table 1.

**Table 1:** Spectroscopic analyses of the flavonoid compounds isolated from *F. vasta* leaves hydroalcoholic extract

Compound	Physical state	^1^H-NMR data	UV data	MS data
Luteolin	Yellow powder	^1^H-NMR (DMSO-d_6_, 400 MHz): δ ppm 12.9 (1H, s, 5-OH), 7.4 (1H, d, *J* = 8 Hz,, H-6′), 7.38 (1H, d, *J* = 2 Hz, H-2′), 6.85 (1H, d, *J* = 8 Hz, H-5′), 6.6 (1H s, H-3), 6.4 (1H, d, *J* = 2 Hz, H-8), 6.15 (1H, d, *J* = 2 Hz, H-6)..		EI-MS: m/z 286
Quercetin	Yellow powder		UV λmax (MeOH): 255, 267, 371; (NaOMe): 270, 320, 420; (AlCl_3_): 270, 455; (AlCl_3_/HCl): 264, 303sh, 315sh, 428; (NaOAc): 257, 274, 318, 383; (NaOAc/H_3_BO_3_): 259, 387.	EI–MS: m/z 302.
Vitexin	Yellow amorphous powder	^1^H-NMR (DMSO-d_6_, 400 MHz): δ 8.04 (d, *J* = 8.5 Hz, 2H, H-2′,6′), 6.88 (d, *J* = 8.5 Hz, 2H, H-3′,5′), 6.42 (s, 1H, H-3), 6.74 (s, 1H, H-6), 4.65 (d, *J* = 9.6 Hz,1H, H-1^″^).	UV λmax (MeOH): 269, 331; (NaOMe): 279, 325 (sh), 391; (AlCl_3_): 276, 303 (sh), 346, 382; (AlCl_3_/HCl): 277; 303, 343, 380 (NaOAc): 278, 387 (NaOAc/H_3_BO_3_): 270, 319, 346.	ESI-MS *m/z*: 433 [M + H]^+^.
Quercetin 3-*O-β*-galactoside	Yellow crystals	^1^H-NMR (DMSO-d_6_, 400 MHz): δ 7.78 (1H, dd, *J* = 2, 8.5 Hz, H-6′), 7.54 (1H, d, *J* = 2 Hz, H-2′), 6.82 (1H, d, *J* = 8.5 Hz, H-5′), d 6.42 (1H, d, *J* = 2 Hz, H-8), 6.24 (1 H,d, *J* = 2 Hz, H-6), 5.5 (1H, d, *J* = 7.5 Hz, H-1″).		(−)ESI-MS: m/z 463 [M-H]^−^.
Quercetin 3-O-rutinoside (Rutin)	Yellow powder	^1^H-NMR (400 MHz, DMSO-d_6_): δ ppm 7.54 (2H, m, H-2′/6′), 6.85 (1H, d, *J* = 9 Hz, H-5′), 6.38 (1H, d, *J* = 2.5 Hz, H-8), 6.19 (1H, *J* = 2.5 Hz, H-6), 5.35 (1H, d, *J* = 7.5 Hz, H-1″), 4.39 (1H, s, H-1″‘), 3.90–3.20 (m, remaining sugar protons), 0.99 (3H, d, J = 6 Hz, H-6″‘).	UV λmax (MeOH): 258, 269, 361; (NaOMe): 276, 322, 416; (AlCl_3_): 232, 276, 302, 366; (AlCl_3_/HCl): 232, 276, 302, 366; (NaOAc): 284, 306, 381; (NaOAc/H_3_BO_3_): 261, 312, 376.	

#### Identification of phenolic compounds by HPLC-PDA/ESI-MS

**Fig. 1:**
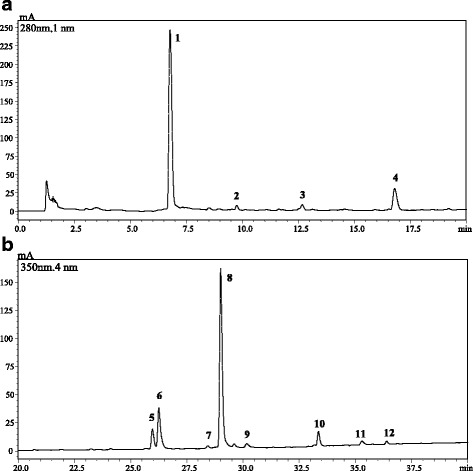
HPLC-PDA chromatograms of the phenolic compounds, extracted at 280 nm (**a**) and 350 nm (**b**) wavelengths, of *F. vasta* leaves hydroalcoholic extract. For peak identification, see Table 2

The quali-quantitative characterization of the phenolic compounds present in the *F. vasta* leaves extract was accomplished by HPLC-PDA/ESI-MS. Baseline compound separation was achieved on the employed fused-core C18 stationary phase; as far as detection is concerned on-line coupling to PDA and MS detection provided complementary information for reliable identification purposes. The analysis revealed the presence of 12 compounds, 2 out of them belonging to the group of phenolic acids (77.09 mg/g extract) and 10 to flavonoids (135.98 mg/g extract). The flavonol quercetin-3-galactoside was found to be the main phenolic compound detected in the extract (81.5 mg/g ± 0.88% RSD), followed by gallic acid (76.36 mg/g ± 2.70% RSD) and isoquercitrin (22.5 mg/g ± 2.02% RSD) (Fig. 1, Table 2).

**Table 2:** HPLC-PDA/ESI-MS (negative ionization mode) polyphenolic fingerprint of *Ficus vasta* leaves extract

No.	t_R (min)_	Molecular Formula	[M-H]^−^	UV/Vis (nm)	Compound	Class	mg/g extract	RSD (%)
1	6.8	C_7_H_6_O_5_	169, 125	210, 270	Gallic acid	Benzoic acid-like acid	76.36	2.70
2	9.7	C_15_H_14_O_6_	289	205, 278	Catechin	Flavan-3-ol-like	6.53	1.87
3	12.6	C_16_H_18_O_9_	353, 191	215, 325	Chlorogenic acid	Cinnamic acid-like	0.73	1.73
4	16.8	C_15_H_12_O_5_	271	283	Naringenin	Flavanone-like	5.84	1.30
5	26.0	C_27_H_30_O_16_	601, 301	254, 354	Rutin	Flavonol-glycoside-like	9.33	1.38
6	26.2	C_21_H_20_O_12_	463, 301	254, 354	Isoquercitrin	Flavonol-glycoside-like	22.50	2.02
7	28.4	C_27_H_32_O_14_	579, 271	254, 354	Naringin	Flavanone-glycoside-like	1.20	3.56
8	29.0	C_21_H_20_O_12_	463, 301	257, 354	Quercetin-3-galactoside	Flavonol-glycoside-like	81.75	0.88
9	30.2	C_21_H_20_O_10_	431, 269	270, 330	Vitexin	Flavone-glycoside-like	0.64	2.95
10	33.4	C_21_H_20_O_11_	447, 285	256, 346	Kaempferol-3-glucoside	Flavonol-glycoside-like	6.72	4.93
11	35.3	C_15_H_10_O_7_	301	370	Quercetin	Flavonol-like	0.98	0.81
12	36.4	C_15_H_10_O_7_	285	265, 365	Luteolin	Flavone-like	0.49	3.31

Column: Ascentis Express C_18_, 15 cm × 4.6 mm, 2.7 μm d.p. (ESI, negative ionization mode; when observed, secondary fragment ions are reported).Values are expressed as the mean ± S.D. (n = 3)

### Antioxidant activity

#### Free radical scavenging activity

**Fig. 2:**
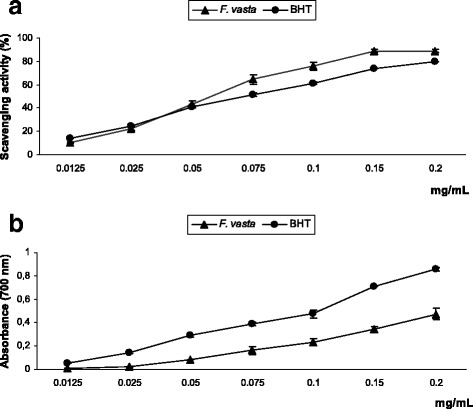
Free radical scavenging activity (DPPH test) (**a**) and reducing power (**b**) of *F. vasta* leaves hydroalcoholic extract. Values are expressed as the mean ± SD (*n* = 3)

The results of DPPH assay are shown in Fig. 2a. *F. vasta* extract displayed strong radical scavenging effect, dose-dependent, which reached about 90% inhibition at the concentration of 0.15 mg/mL. The activity of the extract was higher than that of the standard BHT, as indicated also by the IC_50_ values (0.0672 ± 0.0038 mg/mL and 0.0821 ± 0.0009 mg/mL, respectively).

#### Measurement of reducing power

*F. vasta* extract exhibited reducing power, that increased in a dose-dependent manner; the activity resulted lower than that of BHT, as confirmed by ASE/mL values (3.65 ± 0.48 ASE/mL and 1.97 ± 0.08 ASE/mL) (Fig. 2b).

#### Ferrous ions (Fe^2+^) chelating activity

In the Fe^2+^ chelating activity assay *F. vasta* extract showed mild, dose-dependent, effect (data not shown). As confirmed by the IC_50_ values, the chelating ability of the extract resulted much lower than that of the standard EDTA (0.801 ± 0.007 mg/mL and 0.0067 ± 3.98 E-05 mg/mL, respectively).

#### Protective effect on *Escherichia coli* under peroxide stress

**Fig. 3:**
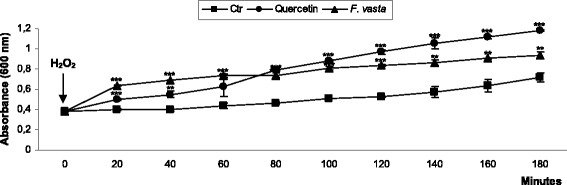
Protective effect of *F. vasta* leaves hydroalcoholic extract on *Escherichia coli* growing under peroxide stress. Values are expressed as the mean ± SD (n = 3). Statistical differences compared to control group with H_2_O_2_ treatment (Ctr + H_2_O_2_) are noted with asterisk (**P < 0.001, ***P < 0.0001)

In a preliminary experiment we established that *F. vasta* extract does not inhibit the growth of *E. coli* at the dose of 1 mg/mL under the experimental conditions utilized in this protocol, thus we tested the extract at the concentration of 1 mg/mL to evaluate its protective ability against the bacteriostatic and bactericidal effects of H_2_O_2_. As shown in Fig. 3, *F. vasta* extract displayed noticeable protective effect on *E. coli* growth under oxidative stress. Addition of 2 mM H_2_O_2_ resulted in a 60-min growth arrest of *E. coli* into the Ctr group. In the culture pretreated with quercetin (0.2 mM), addition of H_2_O_2_ did not inhibit bacterial growth. The pretreatment with *F. vasta* extract (1 mg/mL) provoked a strong protection against H_2_O_2_-induced damage, statistically significant at all time points compared to Ctr group treated with H_2_O_2_ (*P* < 0.001 and *P* < 0.0001). Further, the cell growth of the culture treated with *F. vasta* extract notably exceeded that of quercetin group at 20, 40 and 60 min.

**Fig. 4:**
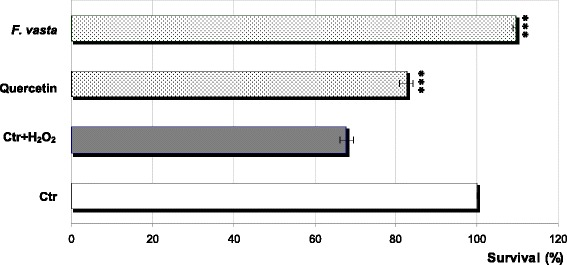
Protective effect of *F. vasta* leaves hydroalcoholic extract on *Escherichia coli* survival under peroxide stress. Values are expressed as the mean ± SD (n = 3). Statistical differences compared to control group with H_2_O_2_ treatment (Ctr + H_2_O_2_) are noted with asterisk (***P < 0.0001)

The results of protective effect on *E. coli* survival are shown in Fig. 4. After 30 min, an elevated loss of viability in the Ctr culture treated with 10 mM H_2_O_2_ (approximately 68% survival) compared to untreated Ctr was observed. In the culture pretreated with *F. vasta* extract, high survival (approximately 110%) was maintained in the presence of 10 mM H_2_O_2_, statistically significant compared to Ctr culture treated with H_2_O_2_ (*P* < 0.0001); even in this case the observed effect was higher than that of quercetin.

#### *Artemia salina* lethality bioassay

*F. vasta* extract did not display any toxicity against brine shrimp larvae (LC_50_ > 1000 μg/mL).

### Antimicrobial activity

The antimicrobial properties of *F. vasta* extract were tested against a representative set of Gram-positive and Gram-negative bacterial strains and the yeast *C. albicans*, according to the protocols recommended by the Clinical and Laboratory Standards Institute [19, 20]. The MIC values of *F. vasta* extract are shown in Table 3.

**Table 3:** The MIC values of *F. vasta* leaves extract

**Gram positive bacteria**	**MIC (μg/mL)**
*Bacillus subtilis* ATCC 6633	> 500
*Listeria monocytogenes* ATCC 13932	125.0
*Staphylococcus aureus* ATCC 29213	62.5
*Staphylococcus epidermidis* ATCC 12228	62.5
**Gram negative bacteria**	
*Escherichia coli* ATCC 10536	250.0
*Escherichia coli* ATCC 25922	250.0
*Pseudomonas aeruginosa* ATCC 15442	> 500
*Salmonella typhimurium* ATCC 13311	250.0
*Salmonella enterica* (Wild type)	250.0
**Yeast**	
*Candida albicans* ATCC 10231	> 500

After 24 h of exposure [20], the extract was effective against almost all the bacteria tested, with the exception of *B. subtilis* and *P. aeruginosa* (MIC > 500 μg/mL). The extract showed higher efficacy towards Gram-positive than Gram-negative bacteria, with *S. aureus* and *S. epidermidis* being the most sensitive strains (MIC: 62.5 μg/mL). None of the tested strains were inhibited by DMSO (maximum 0.5% *v*/v), used as negative control (data not shown). The MBC values indicated that the inhibitory effect of *F. vasta* extract was bacteriostatic within the concentrations tested (MBC >  500 μg/mL). Finally, no activity was detected against *C. albicans* (MIC > 500 μg/mL).

## Discussion

In this work we report for the first time the quali-quantitative characterization of the phenolic compounds present in the hydroalcoholic extract obtained from *F. vasta* leaves collected in Egypt. HPLC-PDA/ESI-MS analysis revealed the presence of phenolic acids and flavonoids.

Phenolic compounds are well known for their antioxidant properties by acting either as free radical scavengers, reducing agents or metal chelators [24]. Flavonoids and phenolic acids represent the largest classes of plant phenolics; phytochemicals from these classes were found to have excellent antioxidant activity in both in vitro and in vivo investigations [25].

Antioxidant activity, especially of phytocomplexes, cannot be evaluated satisfactorily by a simple antioxidant test, but it is strongly suggested the use of various methods in order to acquire a more complete antioxidant profile. In these assays, plant extracts are generally assessed for their function as reducing agents, hydrogen donors, singlet oxygen quenchers or metal chelators, after which they are classified as primary (chain-breaking) and secondary (preventive) antioxidants [25]. Thus, three in vitro assays based on fundamentally different approaches and mechanisms were used to screen the antioxidant potential of *F. vasta* extract: the primary antioxidant properties were examined using the DPPH and the reducing power assays, and the secondary antioxidant ability was determined by measuring the Fe^2+^ chelating activity. Antioxidants can deactivate radicals by two major mechanisms: hydrogen atom transfer (HAT) and single electron transfer (SET); for DPPH test recently a combination of these two reactions, HAT and SET, was suggested to occur, whereas reducing power is recognized as electron transfer based method [25–27].

The results of antioxidant tests showed that *F. vasta* extract possesses primary antioxidant properties; these effects could depend mainly on the presence of flavonoids and phenolic acids, particularly quercetin-3-galactoside and gallic acid, which are present in high amounts in the extract. Previous studies demonstrated the strong radical scavenging properties of these compounds, as well as their ferric reducing ability [28–30]. Abdelwahed et al. [31] demonstrated that gallic acid adopt a hydrogen donating mechanism to scavenge the DPPH radical and it is even more effective than Vitamin E.

In order to investigate the antioxidant efficacy of *F. vasta* extract in a biological setting, the ability to protect bacterial growth and survival from the oxidative stress induced by hydrogen peroxide (H_2_O_2_) was evaluated on *Escherichia coli.* This microbial model is utilized as an effective system to establish the antioxidant properties of medicinal plant extracts or pure compounds [32]; it is easier in terms of experimental operation, lower in cost compared to cellular antioxidant activity assays, and more biologically relevant than the in vitro measurements of antioxidant activity.

The obtained results showed that *F. vasta* extract displayed noticeable protective effects on *E. coli* growth under oxidative stress. These results are similar to those previously reported for different extracts of *Potentilla fruticosa* L., tested at the same concentration of *F. vasta* extract and under the same experimental conditions [33]. Oktyabrsky et al. [34] previously demonstrated the protective effect of several plant extracts on *E. coli* survival in the presence of high concentrations of H_2_O_2_ (10 mM); from a comparison of the results *F. vasta* extract showed a greater activity, displaying total protection against oxidative damage.

The protective effects of some polyphenols, as quercetin and catechin, on growth and survival of *E. coli* under peroxide stress has been previously reported [19]; thus, it can be hypothesized that the polyphenols contained in *F. vasta* extract are the main responsible for the observed activities.

In order to achieve a safe treatment with plant products, numerous research studies have recently been focused on the toxicity of medicinal plants. The brine shrimp (*Artemia salina* Leach) lethality bioassay has been established as a safe, practical and cheap method employed for preliminary assessment of toxicity and have been used for detection of fungal toxins, plant extract toxicity, heavy metals and pesticides [21]. According to Clarkson’s toxicity criterion for the toxicity assessment of plant extracts, those with LC_50_ above 1000 μg/mL are considered as non-toxic [35]. *F. vasta* extract was found to be non-toxic against brine shrimps.

In the last decade, there has been growing interest in the use of plant extracts with low toxicity as sources of natural antimicrobial substances; particularly, the antimicrobial properties of plant extracts containing phenolic compounds were described [36–38].

*F. vasta* extract extract exhibited bacteriostatic activity against almost all the Gram-positive and Gram-negative bacteria tested, particularly against *S. aureus* and *S. epidermidis*.

Our results disagree with those of a previous work, which reported the lack of antibacterial properties of a 80% MeOH extract of *F. vasta* aerial parts against *S. aureus*, *S. epidermidis*, *E. coli* and *P. aeruginosa*, as evaluated by the disk-diffusion method [13]; nonetheless, the extract was tested at the dose of 1.19 μg only, and this could explain the disaccording results.

It can be hypothesized that the antimicrobial properties of *F. vasta* extract could depend on the presence of phenolic compounds. Gram-positive and Gram-negative bacterial species might have different sensitivities against the phenolics contained in *F. vasta* extract because of the difference in their membrane structure and associated cell wall differences. Many of the phenolic compounds were found to be effective against Gram-positive bacteria, whereas they showed no activity or negligible activity against Gram-negative bacteria. The partial hydrophobicity of some phenolic compounds allows them to act efficiently at the membrane-interface of Gram-positive bacteria, this causes the loss of membrane integrity and the dissipation of the proton motive force [39].

It is known that both flavonoids and phenolic acids are effective antimicrobial agents against a wide array of microorganisms [5, 40]. Liu et al. [41] previously reported that quercetin and luteolin showed a broad antimicrobial spectrum of activity on microorganisms including bacteria and fungi, whereas the glycoside derivatives such as quercetin 3-*O*-β-D-glucoside (isoquercitrin) exhibited relatively weak antimicrobial activity. Other authors showed that quercetin 3-*O*-glucoside didn’t display any antibacterial efficacy [42].

The antibacterial activity of some phenolic acids such as gallic against Gram-positive (*S. aureus* and *L. monocytogenes*) and Gram-negative bacteria (*E. coli* and *P. aeruginosa*) was demonstrated; these compounds were found to be more efficient against the reported bacteria than conventional antibiotics such as gentamicin and streptomycin [43]. It was reported that gallic acid could restrain the growth of many bacteria, including methicillin-sensitive *S. aureus*, MRSA, *E. coli*, *P. aeruginosa*, and *S. typhi* [44]. Based on these statements, gallic acid, contained in high amount in the extract, could be the main component responsible of the observed effects.

## Conclusions

This study is the first report on the characterization of the phenolic profile and the evaluation of antioxidant and antimicrobial activities of the leaves of *Ficus vasta* Forssk. growing in Egypt. The results of our investigations showed that *F. vasta* extract possesses strong primary antioxidant properties, as well as antibacterial efficacy, particularly against the Gram-positive tested strains. Besides, the extract showed no toxicity against brine shrimp larvae.

These findings contribute to an increase in knowledge about this species, demonstrating the potential of *Ficus vasta* leaves as safe sources of natural antioxidant and antimicrobial compounds.

## Abbreviations

ACN: acetonitrile
ASE: ascorbic acid equivalent
BHT: Butylated Hydroxytoluene
CFU: colony forming unit
DMSO: dimethyl sulfoxide
DPPH: 2,2-diphenyl-1-picrylhydrazyl
EDTA: Ethylenediaminetetraacetic acid
H_2_O_2_: hydrogen peroxide
IC_50_: mean 50% Inhibitory Concentration
LB: Luria-Bertani
LC_50_: median lethal concentration
MBC: minimum bactericidal concentration
MeOH: methanol
MFC: minimum fungicidal concentration
MHB: Müeller Hinton Broth
RSD: relative standard deviation
SDA: Sabouraud Dextrose Agar
